# Study of snow cover/depth evolution characteristics in Tianshan region of China based on geographical partition

**DOI:** 10.1038/s41598-023-29494-z

**Published:** 2023-02-11

**Authors:** Wei Qiao, Liangfu Xie, Jiabing Zhang, Yongjun Qin, Xuejun Liu

**Affiliations:** 1 College of Civil Engineering and Architecture, Xinjiang University, Urumqi, 830046 China; 2 Xinjiang Civil Engineering Technology Research Center, Urumqi, 830046 China; 3 Xinjiang Academy of Architectural Science (Limited Liability Company), Urumqi, 830002 China

**Keywords:** Environmental impact, Geomorphology

## Abstract

Based on the digital elevation data, snow depth and snow cover remote sensing data, this paper divides six snow evolution areas and geographical partitions, extracts the geographical partitions of each evolution area and obtains the geographical characteristics of the evolution area for analysis. The results show that: (1) From 2003 to 2017, the average snow area decreased at a rate of − 0.004, and the average snow depth increased at a rate of 0.03. (2) The snow in the middle altitude hill with shady gentle slope area is the most obvious in the seasonal evolution, and the percentage of this region in the seasonal snow evolution area is 5.46%, the snow depth in the middle altitude hill with sunny and gentle slopes area increased and decreased significantly in the past 15 years, and the percentage of this region in the SD significant changes evolution area was 6.32%. The snow in the low relief middle altitude mountain with shady and moderate slope area not only shows obvious seasonal evolution, but also increases and decreases significantly in snow depth. And the percentage of this region in the seasonal snow significant evolution area is 5.82%. (3) The geographical partitions with the largest area in all evolution areas is the middle altitude hill with sunny and gentle slopes area (4.75%). (4) The geographical partition with the largest variation of snow depth in Tianshan region is the low relief middle altitude mountain with shady and moderate slope area (12.02 cm). (5) The snow accumulation and melting are obvious in the range of 1000–3500 m above altitude, different geomorphology types lead to obvious differences in snow characteristics. The snow melting is most obvious in the gentle slope area of the low topographic relief geomorphology types, and the snow accumulation is most obvious in the steep slope area of the middle relief geomorphology types.

## Introduction

Snow in Tianshan region is an important source of water resources in Xinjiang, and the spatial distribution and changes of snow are influenced by topography and climate change^1–3^, while Tianshan region in Xinjiang is the most typical representatives of large mountain ecosystems in the global temperate arid regions, glacial floods, snowmelt floods, glacial mudslides, ice avalanches and other snow and ice disasters caused by snow evolution in Xinjiang pose a major threat to local residents, economy and national defense^4–6^, In addition, the study of snow evolution characteristics in Tianshan region can provide theoretical guidance for the rational development and utilization of water resources in Xinjiang, which is of great significance to ensure the safe supply of water resources in Xinjiang. Therefore, it is urgent and necessary to study the snow evolution in Tianshan region. Many scholars have studied the evolutionary characteristics of the snowpack and have obtained a large number of results^7,8^, Liston^9^ established a mathematical relationship between three characteristics of snow water equivalent (SWE), snowmelt rate, and snow cover recession area to describe the seasonal snowmelt process from late winter to spring melt time. Letsinger et al.^10^ developed a distributed energy balance model to simulate the evolution and ablation of snow fields in rugged terrain. Martin Moreno et al.^11^ studied the evolution characteristics of snow cover in 75° north latitude Spitsbergen area and found that the effect of terrain and wind on snow cover is the main factor affecting the distribution of snow cover. The research results show that the plains and valleys are more prone to snow cover, while the slopes are more difficult to produce snow cover. Seasonally, the snow cover melts faster in spring, which is more likely to produce avalanches and form new landforms. Chen et al.^12^ used six surface models to simulate the one-year snow water equivalent (SWE) in the headwaters of the Colorado River and validated them against weather station data, showing that all models are consistent with the seasonal evolution of measured snow water equivalent data. Cantet et al.^13^ used particle filtering in combination with manually measured snow water equivalent data to improve the temperature and rain-snow model, and compared the improved model estimates with the spatial estimates of snow survey data, and found that particle filtering could better improve the accuracy of the spatial distribution of snow water equivalent. Baba et al.^14^ combined MERRA-2 and ERA5 satellite data to analyze the spatial distribution of snow water equivalent in the High Atlas region, and the results show that the snow water equivalent distribution obtained from the combination of the two satellites performs well in the snowpack area during most of the hydrological year. Zhang et al.^15^ extracted glacier and snow cover areas from MODIS data and studied the relationship between glacier evolution trends and snow cover evolution in the Tarim Basin.

In the current study of snow in the Tianshan region, more scholars have discussed the interaction between snow and climate factors^16–20^. Li et al.^21^ quantified climatic snow parameters from 48 meteorological stations in the Central Asian Tianshan from 1961 to 2015, analyzed the effects of seasonal temperature and precipitation on snow accumulation and melting, and found that the maximum snow depth and the number of days of snow accumulation showed similar distributions in space. Qin et al^22^ studied the relationship between snowfall and climate change in the Tianshan region and the mechanism using meteorological station data. Hanati et al.^23^ studied the snow melting pattern in Tianshan Mountains, the characteristics of permafrost hydrothermal changes, and their effects on temperature and snow melt. Sheng et al.^24^ found that snow albedo in the Tianshan and northern Xinjiang regions was affected temporally by temperature changes and spatially by pollutants, which had the greatest effect on snow albedo during the snow stabilization and melting periods.

In summary, there are many factors influencing snow evolution in the Tianshan region of Xinjiang, and the research results have certain reference value, the research content is mainly about the influence of climate factors such as temperature and precipitation on snow evolution, while in terms of the influence of geographical factors on snow evolution, most scholars only analyze the effects of single geographical factors on snow depth, snow cover and snow days, while the relationship between the zoning of multiple geographical factors combined with snow evolution is less considered. Based on this, this paper takes the Tianshan region of Xinjiang as the study area and uses the satellite data of snow accumulation from 2003 to 2017, combined with different geographical divisions, to analyze the characteristics of snow accumulation evolution under geographical divisions in the Tianshan region of Xinjiang.

## Study area

**Figure 1:**
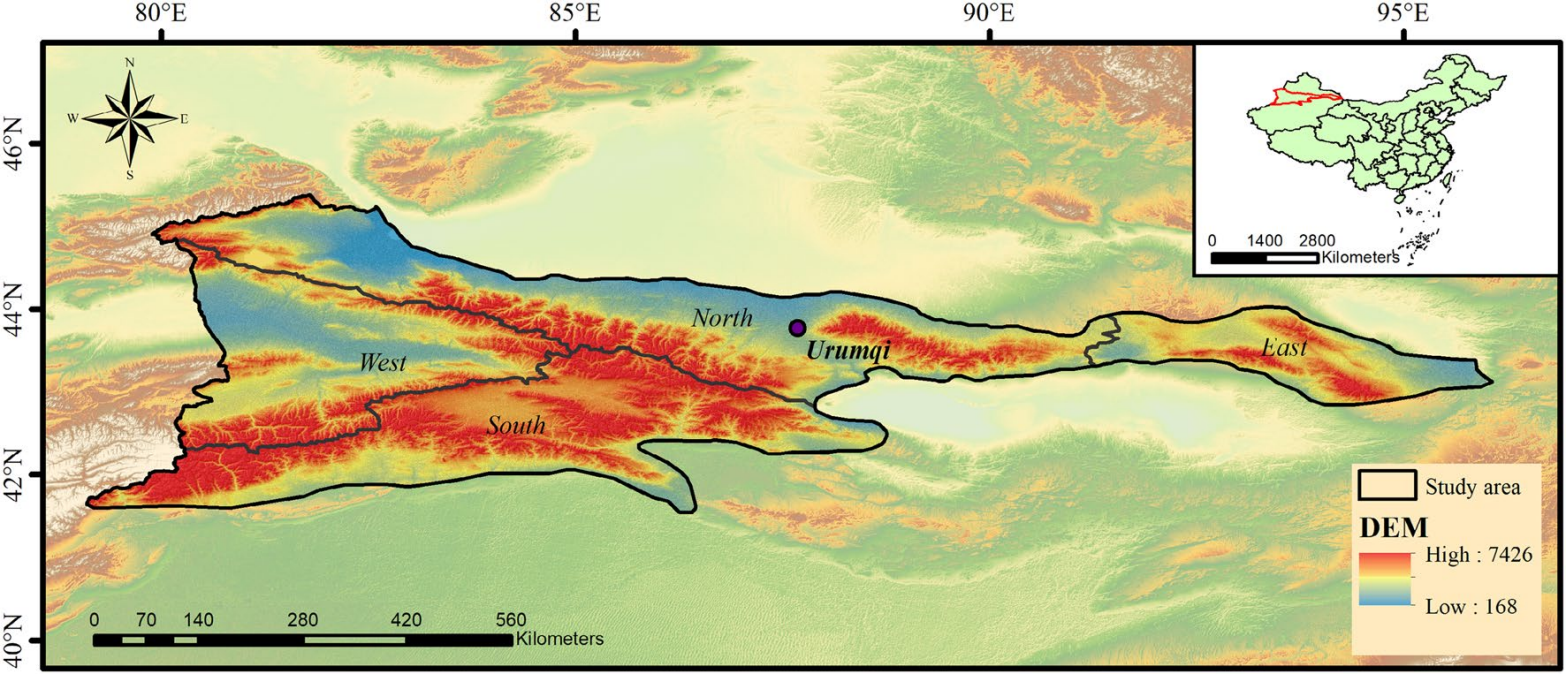
Study area. The maps were generated by ArcGIS 10.2, URL: http://support.esri.com/Products/Desktop/arcgis-desktop/arcmap/10-2-2#overview.

The Tianshan Mountains are located in the hinterland of the Eurasian continent. The mountains run east–west, starting from Uzbekistan in the west and extending to Kyrgyzstan and northwestern China and southwestern Mongolia in the east, with a total length of about 2500 km, it is the largest independent latitudinal mountain system in the world^25,26^. The study area is the Tianshan Mountains in China region (Tianshan region, Xinjiang, China)^27,28^, located in the hinterland of the Xinjiang Uygur Autonomous Region, west to Yili Prefecture, east to Hami City, north to Bo Prefecture, and south to Aksu Region, Geographic location is 79E–96E, 41N–45N, with an area of 237,253.21 km^2^. The Tianshan region (study area) accounts for two-thirds of the length of The Tianshan Mountains and is about 1700 km long and 350 km wide^29^. The topography of the study area is high in the west and low in the east, with wide mountains, average altitude of 2181 m, temperate continental climate, and a maximum snow area of 8940.24 km^2^. The study area is divided into four regions according to the district boundary of Xinjiang Uygur Autonomous Region (Fig. 1).

## Data and methods

### Data

1. Digital Elevation Model Data (DEM)

Digital Elevation Model data from the NASA (https://earthdata.nasa.gov/) ASTER GDEM V3, coordinate system is UTM/WGS84, spatial resolution is 1 rad/s (about 30 m), data type is GEOTIFF, coverage is global.

(2) Snow remote sensing data

Snow cover data (“An improved Terra–Aqua MODIS snow cover and Randolph Glacier Inventory 6.0 combined product (MOYDGL06*) for high-mountain Asia between 2002 and 2018”)^30^ and snow depth data (Long-term series of daily snow depth dataset over the Northern Hemisphere based on machine learning (1980–2019)^31^ were analyzed during the current study are available in the National Tibetan Plateau Data Center repository, (https://data.tpdc.ac.cn/). Snow cover data is based on (MODIS) Terra and Aqua images, using a de-clouding algorithm to increase accuracy and reduce bias, with a spatial resolution of 500 m, and a total of 46 images of snow parameters for 8 days of synthetic data a year. Snow depth data spatial resolution is 25 km, this data is the daily snow depth data in cm. This paper uses the snow cover and snow depth data from 2003 to 2017.

### Method

#### Snow parameters average calculation

The annual or seasonal averages of snow cover area and snow depth in the study area from 2003 to 2017 were calculated in ArcGIS according to Eq. (1).

1 $$ A = \frac{{\sum\limits_{i = 1}^{n} {X_{i} } }}{n} $$

where A is the average annual or seasonal value of snow cover or snow depth; *i* = 1,2,3…*n*, is an annual or seasonal variable; *X*_*i*_ is the snow cover area percentage or snow depth at time *i*; *n* is the number of time series.

#### Sen’s MK trend analysis

Sen’s slope estimator is a trend calculation method with nonparametric statistics. The method is computationally efficient, insensitive to measurement errors and outlier data, and is often used in trend analysis of long time series data with the following Eq. (2).

2 $$ Sen = median\left( {\frac{{x_{j} - x_{i} }}{j - i}} \right)(j > i,i = 1,2,3, \ldots ,n) $$

where *median* () is the median value of the calculation, *Sen* represents the trend degree; the positive or negative of *Sen* indicates that the snow depth is increasing or decreasing trend, and its value is the degree of change; *x* is the time series (n = 15); *x*_*i*_ and *x*_*j*_. represent snow depth values in the year *i* and *j* respectively.

Mann–Kendall trend test (MK) is a non-parametric time series trend test, which does not require the measured values to obey normal distribution, is not affected by missing values and outliers, and is applicable to the trend significant test of long time series data. Its calculation formula is as follows:

For the sequence *x*_*t*_ = *x*_1_, *x*_2_,…, *x*_*n*_, first determine the magnitude relationship between *x*_*i*_ and *x*_*j*_ in all dual values (*x*_*i*_, *x*_*j*_* j* > *i*) (defined as *S*). The calculation formula of test statistic *S*:

3 $$ S = \sum\limits_{i = 1}^{n - 1} {\sum\limits_{j = i + 1}^{n} {{\text{sgn}} \left( {x_{j} - x_{i} } \right)} } $$

where *sgn*() is the symbolic formula and the calculation formula is as follows:

4 $$ {\text{sgn}} \left( {x_{j} - x_{i} } \right) = \left\{ {\begin{array}{*{20}l} { + 1} \hfill & {x_{j} - x_{i} > 0} \hfill \\ 0 \hfill & {x_{j} - x_{i} = 0} \hfill \\ { - 1} \hfill & {x_{j} - x_{i} < 0} \hfill \\ \end{array} } \right. $$

The trend test is performed using the test statistic Z. The Z value is calculated as follows:

5 $$ Z = \left\{ {\begin{array}{*{20}l} {\frac{S - 1}{{\sqrt {Var(S)} }},} \hfill & {S > 0} \hfill \\ {0,} \hfill & {S = 0} \hfill \\ {\frac{S + 1}{{\sqrt {Var(S)} }},} \hfill & {S < 0} \hfill \\ \end{array} } \right. $$

where *S* is the test statistic; *x*_*i*_ and *x*_*j*_ represent the values of years *i* and *j*, respectively; *n* is the number of samples in the sequence; *sgn*() is the symbolic formula; *Var*(*S*) is the variance of *S*; and Z is the standardized test statistic. When $\left| Z \right| \ge Z_{(1 - \alpha /2)}$, the trend is significant.

Sen’s trend analysis combined with MK test has been widely used in the analysis of meteorological time data, and more research results have been achieved^32–34^. In snow parameters analysis, the Sen-MK method performs well in snow parameters trend analysis over long time spans such as monthly, seasonal and annual^35–37^. Therefore, this paper uses the Sen-MK method for trend analysis of snow depth data to delineate snow depth evolution regions.

#### Geomorphology type division

Topographic relief is the difference between the maximum elevation and the minimum elevation in a range of a specific size. It can describe the topographic characteristics of a range from a macroscopic point of view. After several times of analyses and verification, the best analysis range is 53 × 53 image elements (about 2.56 km^2^), and the topographic relief of the study area is obtained by calculating the raster in ArcGIS according to Eq. (6).

6 $$ Tr = E_{\max } - E_{\min } $$

where *Tr* is the topographic relief, *E*_max_ is the maximum elevation of the analysis range, *E*_min_ is the minimum elevation of the analysis range.

According to the classification of Chinese basic terrestrial geomorphologic types^38^, The elevation and topographic relief of the study area are divided into geomorphologic types and codes. According to Table 1, the elevation is divided into 4 grades and the topographic relief is divided into 7 grades, and 25 geomorphologic types are classified according to the classification.

**Table 1:** Classification of the basic terrestrial geomorphologic types of study area.

Altitude relief	Low altitude (< 1000 m)	Middle altitude (1000–3500 m)	High altitude (3500–5000 m)	Highest altitude (> 5000 m)
Plain (< 30 m)	(11) Low altitude plain	(12) Middle altitude plain	(13) High altitude plain	(14) Highest altitude plain
Platform (30–50 m)	(21) Low altitude platform	(22) Middle altitude platform	(23) High altitude platform	(24) Highest altitude platform
Hill (50–200 m)	(31) Low altitude Hill	(32) Middle altitude hill	(33) High altitude hill	(34) Highest altitude hill
Low relief mountain (200–500 m)	(41) Low relief low altitude mountain	(42) Low relief middle altitude mountain	(43) Low relief high altitude mountain	(44) Low relief highest altitude mountain
Middle relief mountain (500–1000 m)	(51) Middle relief low altitude mountain	(52) Middle relief middle altitude mountain	(53) Middle relief high altitude mountain	(54) Middle relief highest altitude mountain
High relief mountain(10,000–2500 m)	–	(62) High relief middle altitude mountain	(63) High relief high altitude mountain	(64) High relief highest altitude mountain
Highest relief mountain(> 2500 m)	–	–	(73) Highest relief high altitude mountain	(74) Highest relief highest altitude mountain

Geomorphologic type codes are in parentheses.

#### Geographical partition

Based on the DEM data, the slope and aspect distribution of the study area are obtained by ArcGIS spatial analysis, the aspect was graded according to Table 2, and the geographical partition code was calculated by layer superposition using the raster calculator according to the Eq. (7).

7 $$ GP = R_{Geomo} \cdot 100 + R_{Slope} \cdot 10 + R_{Aspect} $$

where *GP* is the geographical partition code, *R* is the geomorphological code, slope and aspect grade.

**Table 2:** Slope and aspect grade.

Slope			Aspect		
Grade	Range	Class name	Grade	Range	Class name
1	0–10.06°	Gentle slope	1	–	Plane
2	10.06°–21.51°	Moderate slope	2	0°–45°, 315°–360°	Shady aspect
3	21.51°–34.34°	Steeper slope	3	45°–135°	Semi-shady aspect
4	34.35°–88.46°	Steepest slope	4	135°–225°	Sunny aspect
			5	225°–315°	Semi-sunny aspect

#### 3.2.5 Graphical abstract

**Figure 2:**
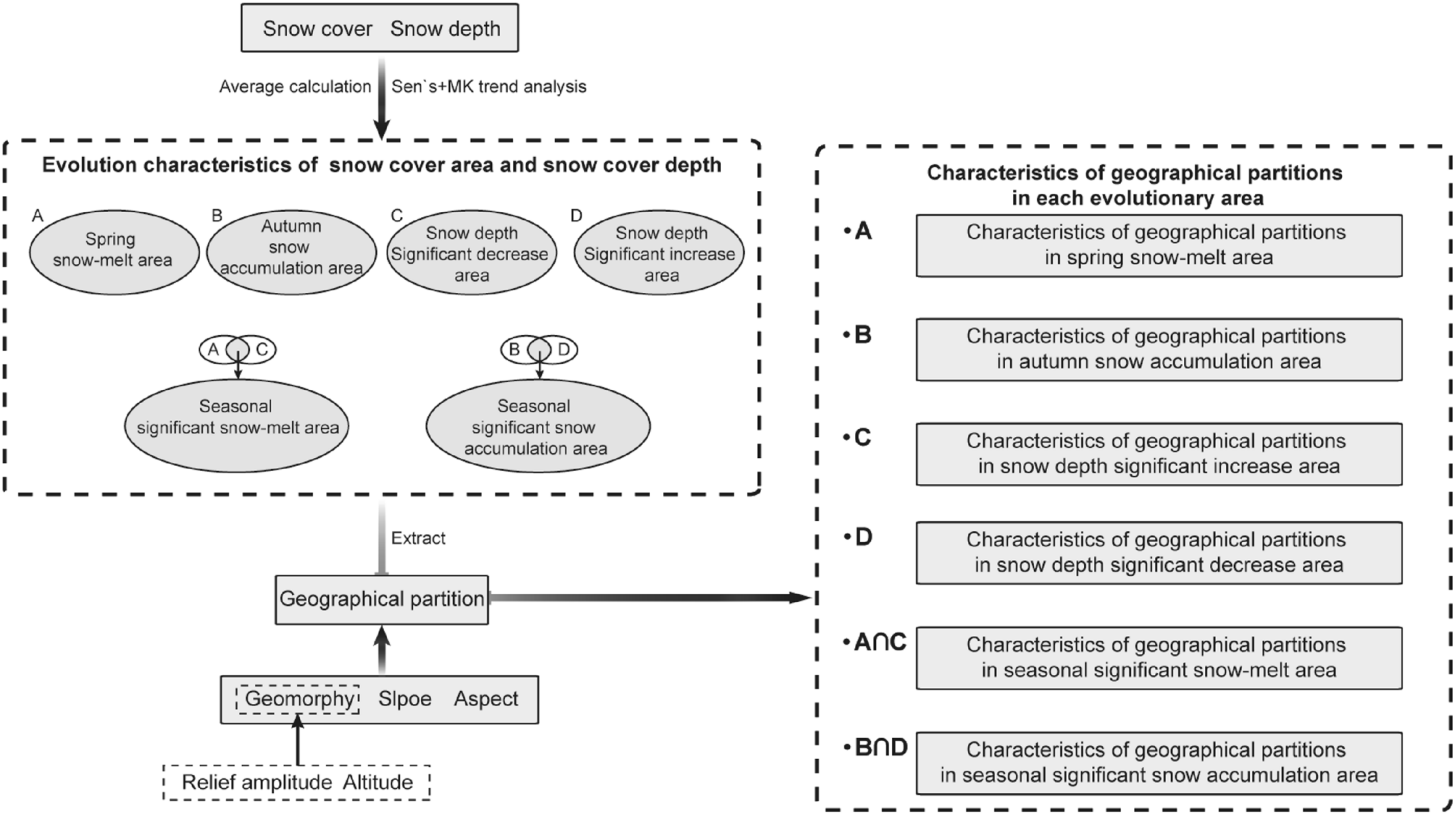
Workflow.

Firstly, the average value calculation and Sen’s MK trend method are used to process the data of snow cover and snow depth, and six snow evolution areas are divided. Then, the geographical partition of Tianshan region is divided based on DEM data, and finally the geographical partition in each snow evolution area is extracted respectively. The entire workflow is shown in Fig. 2. This paper uses snow parameters indices defined as shown in Table 3.

**Table 3:** Snow parameters index and its definition.

Indices	Definition	Unit
Snow cover area percentage (SCP)	The percentage of snow cover area to study area	%
Snow depth (SD)	The average of regional snow depth	cm
Geographical partition area in evolution area percentage (GPEP)	The percentage of geographical partition area and snow evolution area	%
Geographical factor area in evolution area percentage (GFEP)	The percentage of geographical factor area and snow evolution area	%

## Results

### Analysis of the spatial and temporal evolution of SCP/SD

#### Time evolution characteristics of SCP/SD

1. Interannual evolution characteristics of SCP/SD

**Figure 3:**
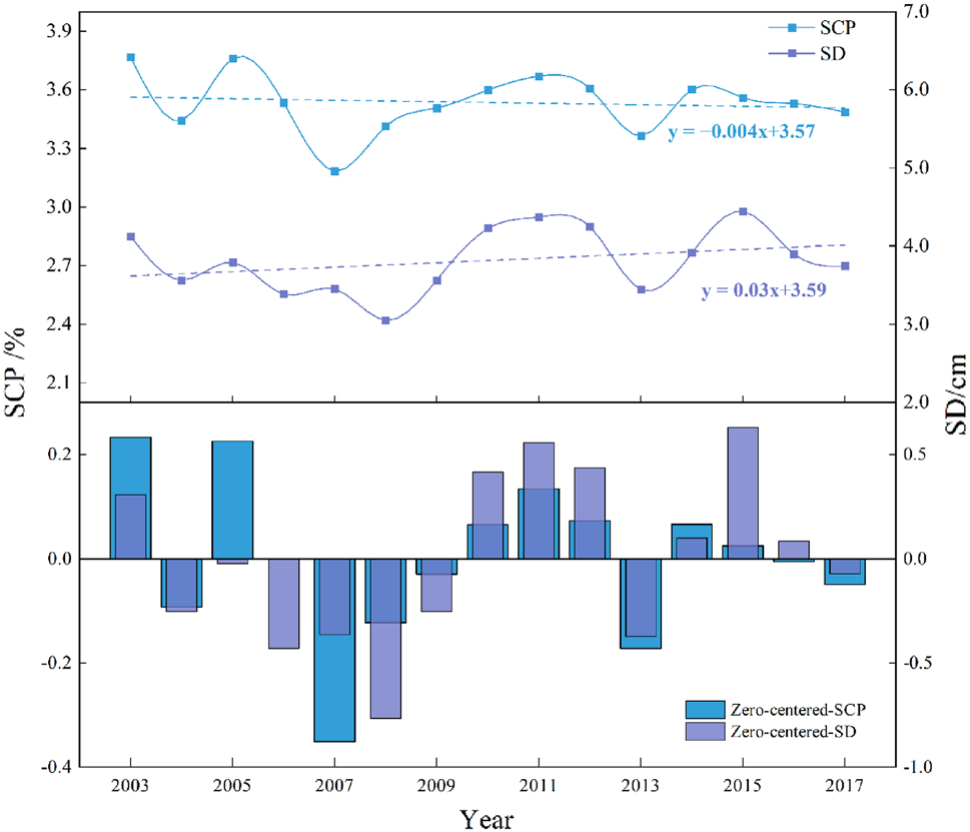
Interannual variation in SCP/SD.

The SCP and SD in the Tianshan region from 2003 to 2017 are shown in Fig. 3, with an overall fluctuating downward trend in snow cover area (rate of decline of 0.004), For example, the SCP decreased from 3.77% to 3.18% from 2003 to 2007, but there was a sudden increase in 2005 (3.76%). SCP increased from 3.18 to 3.67% from 2007 to 2011; SCP decreased from 3.67 to 3.49% from 2011 to 2017, but there was a sudden increase (3.60%) in 2014. The overall SD shows a fluctuating increase (rate of increase of 0.03), For example, from 2003 to 2008, it decreased from 4.12 to 3.05 cm, from 2008 to 2011, it increased from 3.05 to 4.37 cm, from 2011 to 2017, it decreased from 4.37 to 3.74 cm, and in 2015, there was a sudden increase (4.45 cm).

The zero-centered SCP/SD is the difference between the annual average SCP/SD and the fifteen-year average SCP/SD. From the changes of the fifteen-year zero-centered SCP/SD, the maximum zero-centered-SCP was − 0.35% in 2007, the maximum zero-centered-SD was 0.63 cm in 2015, and the zero-centered SCP/SD was smaller in 2004, with a zero-centered-SCP value of − 0.09% and zero-centered-SD value of − 0.25 cm, with a more stable variation.

(2) Seasonal evolution characteristics of SCP/SD

**Figure 4:**
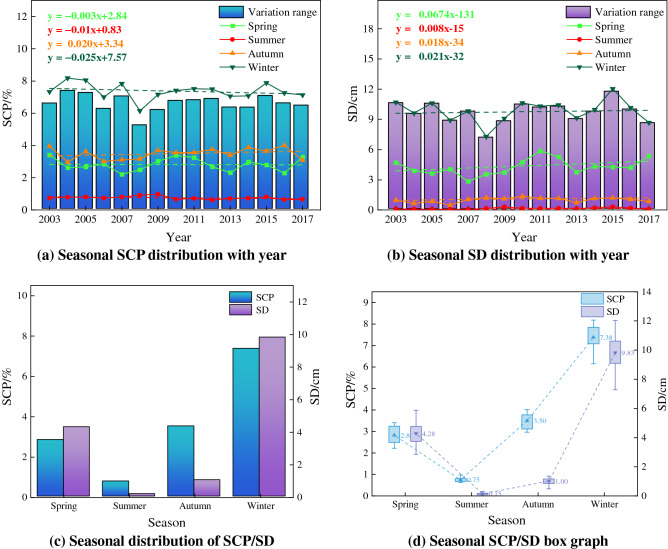
Seasonal SCP/SD statistics chart.

Since the snow cover and snow depth data are recorded at different times, the SCP/SD are divided into seasons according to Table 4 and the area and depth are counted according to four seasons: spring, summer, autumn and winter. The seasonal evolution of snow cover data from 2003 to 2017 is shown in Fig. 4, the SCP from 2003 to 2017 showed a decreasing trend in spring, summer, and winter, with a decrease rate of -0.003, − 0.01, and − 0.025 respectively, and an increase in autumn with a growth rate of 0.02. The SD showed an increasing trend in all four seasons, with growth rates of 0.0674, 0.008, 0.018, and 0.021 respectively. The average change value of SCP/SD in each year is obtained by subtracting the summer SCP/SD from the winter SCP/SD from 2003 to 2017. The SD change in 2015 is the largest (11.74 cm), and the SCP change in 2004 is the largest (7.40%) (Fig. 4a and b). Among the seasonal SCP/SD, SCP and SD are the largest in winter (7.3%/9.83 cm), followed by spring (2.8%/4.28 cm) and autumn (3.5%/1.00 cm), and the smallest in summer (0.7%/0.15 cm) (Fig. 4c and d).

**Table 4:** Seasonal division rules of SCP/SD.

Season	Snow cover data/day	Snow depth data/month
Spring	65–153	3–5
Summer	161–249	6–8
Autumn	257–337	9–11
Winter	345–57 (next year)	12–2 (next year)

#### Spatial evolutionary characteristics of SCP/SD

1. Analysis of the spatial evolution characteristics of SCP

**Figure 5:**
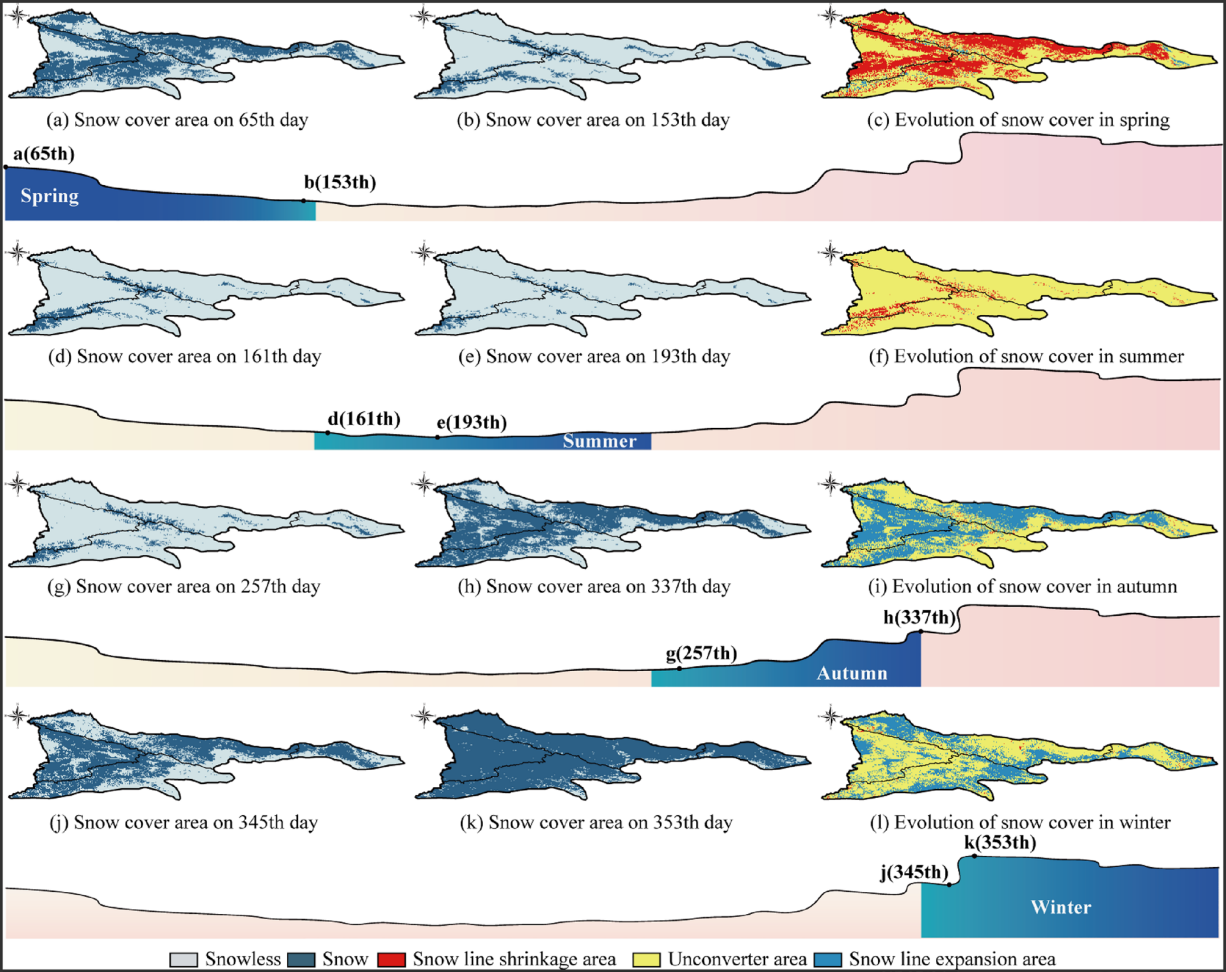
Spatial variation of seasonal snow cover. The maps were generated by ArcGIS 10.2, URL: http://support.esri.com/Products/Desktop/arcgis-desktop/arcmap/10-2-2#overview.

The area of snow cover changed greatly in 2004 compared with other years, and the zero-centered-SCP was smaller. The evolution of snow cover in 2004 was stable in the middle of 15 years. Therefore, the snow cover data in 2004 were analyzed in seasonal order. In Fig. 5, the snowless areas in a, b, d, e, g, h, j, and k are assigned 0, and the snow areas are assigned 1. The layers with the largest representative snow area in the four seasons (Fig. 5a, d, h, k) and the layers with the smallest representative snow area in the four seasons (Fig. 5b, e, g, j). The layers value of the same season is subtracted according to the time sequence of the snow cover evolution curve to obtain seasonal snow cover evolution areas (Fig. 5c, f, i, l). In the seasonal snow cover evolution areas, the value of snow line shrinkage area is − 1, the value of the unchanged area is 0 and the value of snow line expansion area is 1. Spring and summer are the periods of snow melting, autumn and winter are the periods of snow accumulation. Spring is the season with the largest snow melting, and the percentage of ablation area with the study area is 41.12%, which is distributed the piedmont plain of the study area. The snow area in summer is the smallest, and the area percentage between melt area and study area is 5.97%, which is distributed in high altitude perennial snow cover areas in the north, west and south part of the study area. Autumn is the season of maximum snow accumulation, and the percentage of accumulation area with the study area is 48.67%, mainly in the western and northern low altitude areas of the study area. The percentage of winter snow accumulation area to the study area is 36.26%, in autumn most of the study area is covered with snow, so the winter snow accumulation area is mainly distributed in the edge of the study area.

(2) Analysis of the spatial evolution characteristics of SD

The evolutionary trend and significance of snow depth were analyzed by the Sen-MK method, setting $\alpha = 0.05$ significance level , when the absolute value of Z is greater than 1.96, it means that it passed the significance test with 95% confidence level, and the evolutionary trend and significance of the annual and seasonal average snow depth were analyzed by classifying the trend significance according to Table 5

**Table 5:** Sen’s MK trend categories.

Sen	Z	Trend characteristics
Sen > 0	Z > 1.96	Significant increase
	Z ≤ 1.96	Indistinctive increase
Sen = 0	Z	Unchanged
Sen < 0	Z ≤ 1.96	Indistinctive reduce
	Z > 1.96	Significant reduce

**Figure 6:**
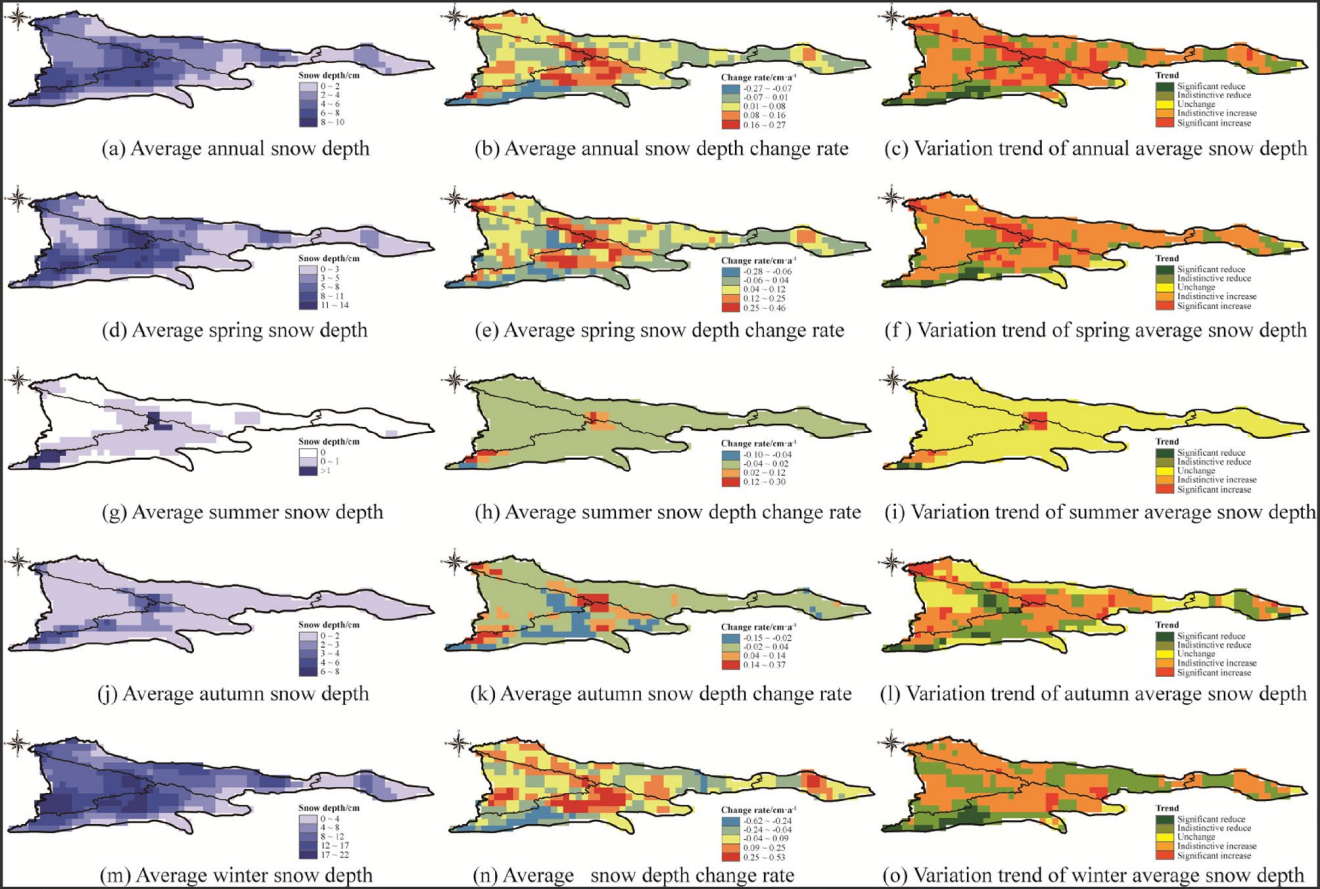
Variation of annual and seasonal SD. The maps were generated by ArcGIS 10.2, URL: http://support.esri.com/Products/Desktop/arcgis-desktop/arcmap/10-2-2#overview.

From Fig. 6, it can be seen that the higher annual SD area (> 6 cm) in the study area is mainly distributed in the central and western part of the study area, the annual SD significant increase area is distributed in the central to northwestern part of the study area with a variation rate interval of 0.08–0.27 cm·a^− 1^, and the annual SD significant decrease area is distributed in the southwestern boundary part of the study area with a variation rate interval of − 0.07 to − 0.27 cm·a^− 1^. The higher SD area (> 8 cm) in spring was mainly distributed in the central and southwestern part of the study area, the spring SD significant increase area was distributed in the central and northwestern part of the study area, with a variation interval of 0.12–0.46 cm·a^− 1^, and a smaller number of the spring SD significant decrease area raster were distributed in the southwestern boundary of the study area, with a variable rate interval of − 0.06 to − 0.28 cm·a^- 1^. The area with snow (> 1 cm) in summer in the study area is mainly the perennial snow area (at the central mountain peak of the study area and the southwest boundary mountain peak), the summer SD significant increase area is sporadically distributed in the perennial snow area with a variable rate range of 0.12–0.30 cm·a^−1^, and the summer SD significant decrease area is distributed in the southwest mountain peak area with a variable rate range of − 0.10 to − 0.04 cm·a^−1^. The higher SD area (> 4 cm) in autumn was mainly distributed in the central to southwestern mountain area, the winter SD significant increase area was distributed in the northwestern part with a rate of change interval of 0.14–0.37 cm·a^−1^, and the winter SD significant decrease area was distributed in the southwestern border part with a rate of change interval of − 0.62 to − 0.24 cm·a^−1^. Overall, the SD in the central part of the study area showed a significant increase, while that in the southwest boundary showed a significant decrease. The SD in the interannual, spring, summer and autumn showed an increasing trend at the rates of 0.07 cm·a^−1^, 0.16 cm·a^−1^, 0.05 cm·a^−1^ and 0.05 cm·a^-1^, respectively, while that in winter showed a decreasing trend at the rate of − 0.09 cm·a^−1^ (Table 6).

**Table 6:** Statistics on the rate of change of significant areas.

Average snow depth	Number of significant area raster	Change rate/cm·a^−1^
Annual	119	0.07
Spring	86	0.16
Summer	20	0.05
Autumn	110	0.05
Winter	48	− 0.09

### Distribution characteristics of geographical partitions in the Tianshan region

**Figure 7:**
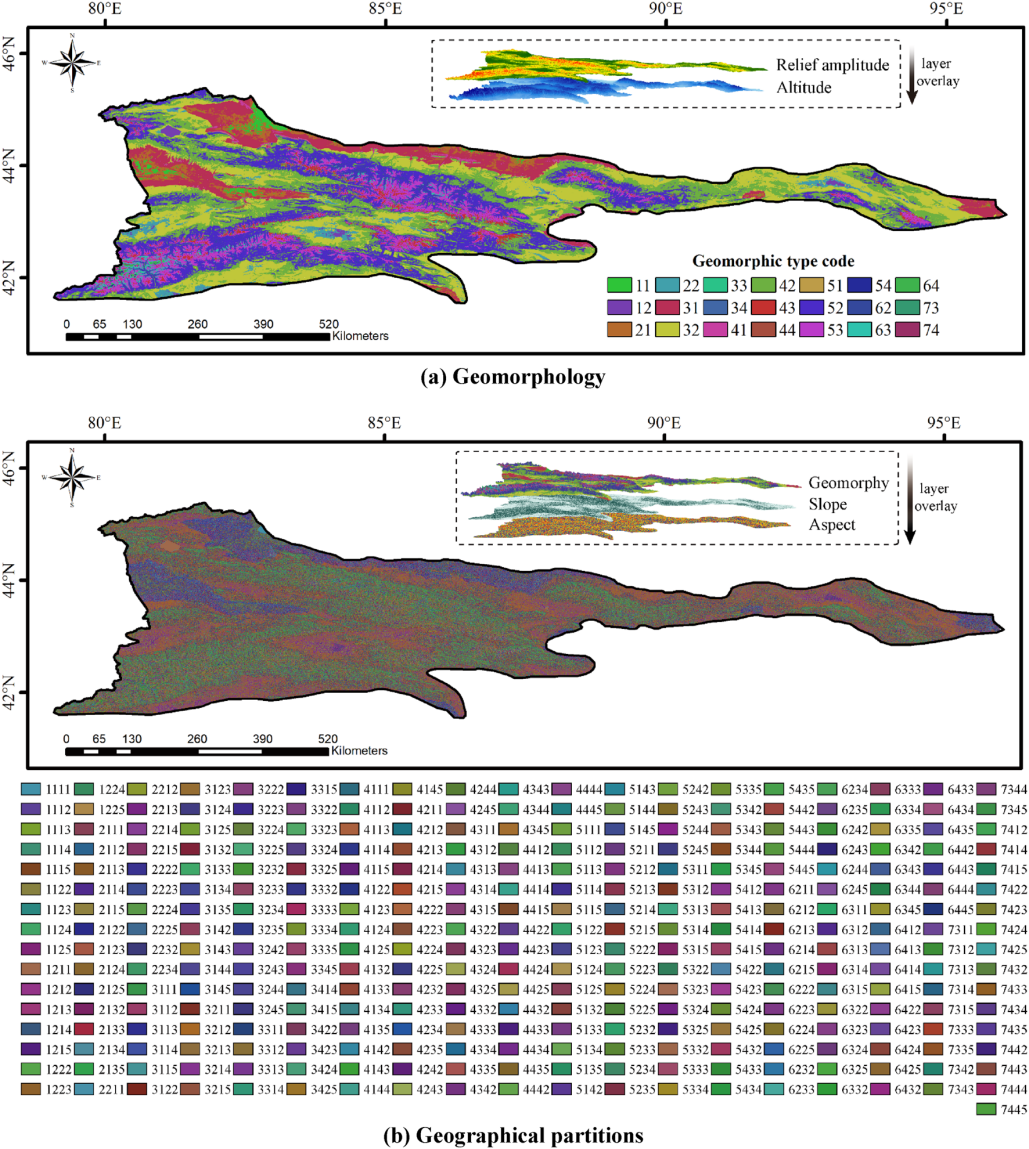
Distribution of geomorphology and geographical partitions in the Tianshan region. The maps were generated by ArcGIS 10.2, URL: http://support.esri.com/Products/Desktop/arcgis-desktop/arcmap/10-2-2#overview.

**Figure 8:**
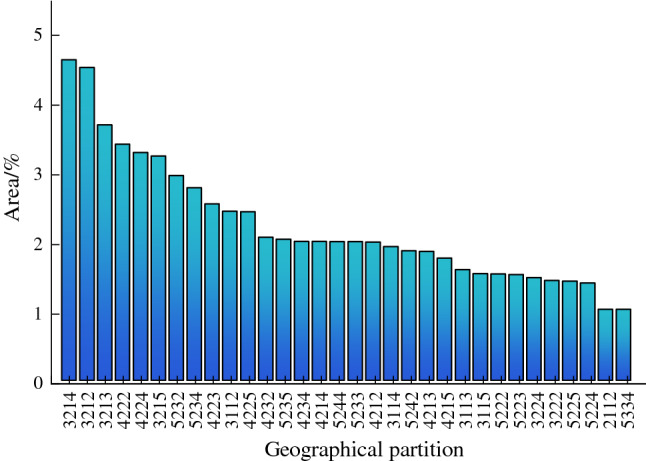
Statistical results of geographical partition.

The geomorphological distribution of Tianshan region is shown by Fig. 7a, mainly low relief middle altitude mountain (42), middle relief middle altitude mountain (52), middle altitude hill (32). The distribution of geographical partitions is shown in Fig. 7b, and the study area is divided into 305 partitions. Ignoring small-area partitions, all kinds of geographical partitions with area ratio greater than 1% in Fig. 7b are counted. The statistical results are shown in Fig. 8: the partitions with area ratio greater than 1% account for 71.75% of the total area, and the single geographical factors with the largest area ratio are middle altitude hill (21.05%), gentle slope (41.83%) and sunny aspect (29.45%), respectively. For geographical partition, the middle altitude hill with sunny and gentle slopes area (3214) occupied the largest proportion (4.65%), followed by the middle altitude hill with shady gentle slope area (3212, 4.53%).

### Geographical partitions distribution characteristics within the snow evolutionary area

**Figure 9:**
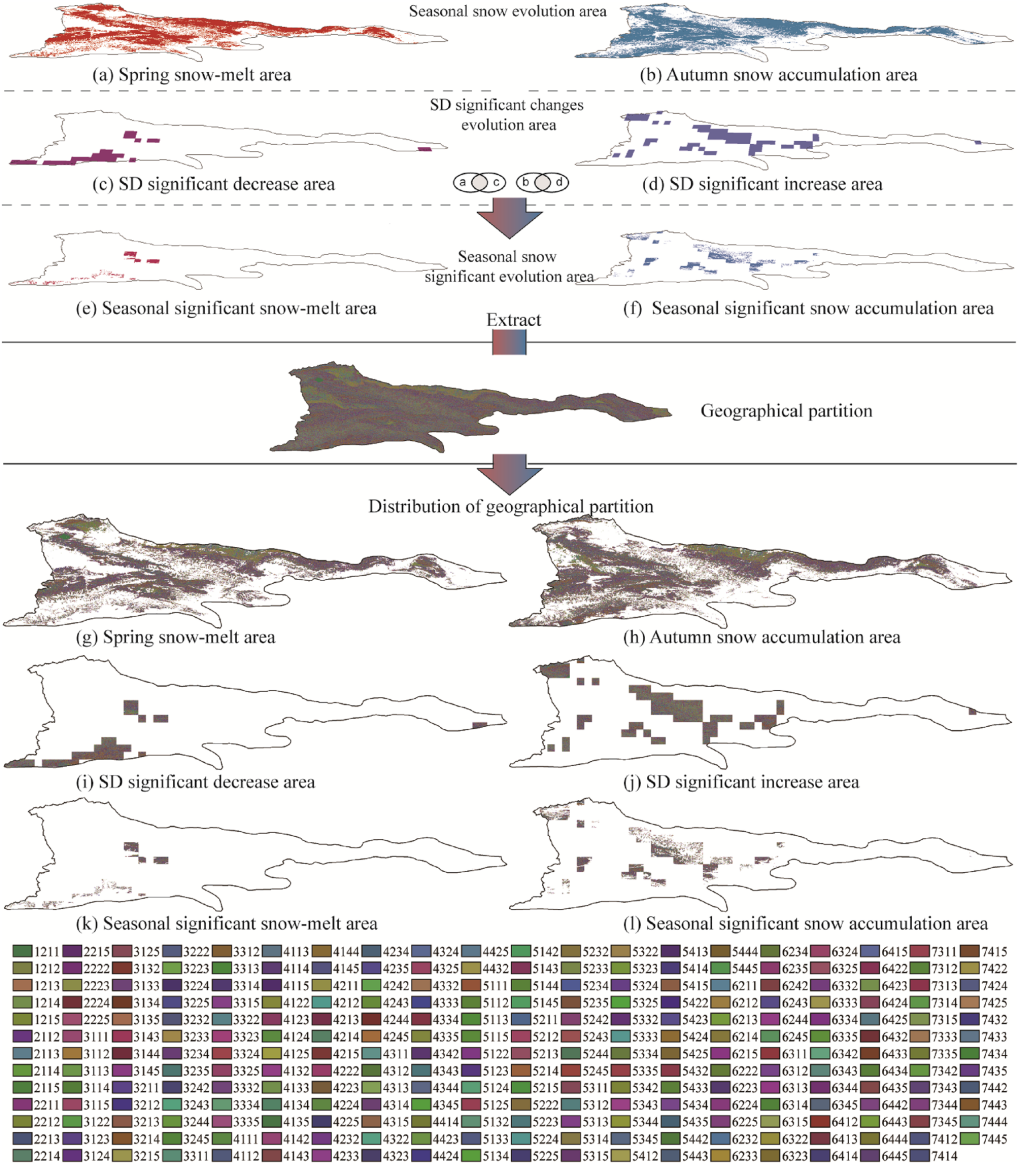
Distribution of snow evolution area and internal geographical partitions. The maps were generated by ArcGIS 10.2, URL: http://support.esri.com/Products/Desktop/arcgis-desktop/arcmap/10-2-2#overview.

**Figure 10:**
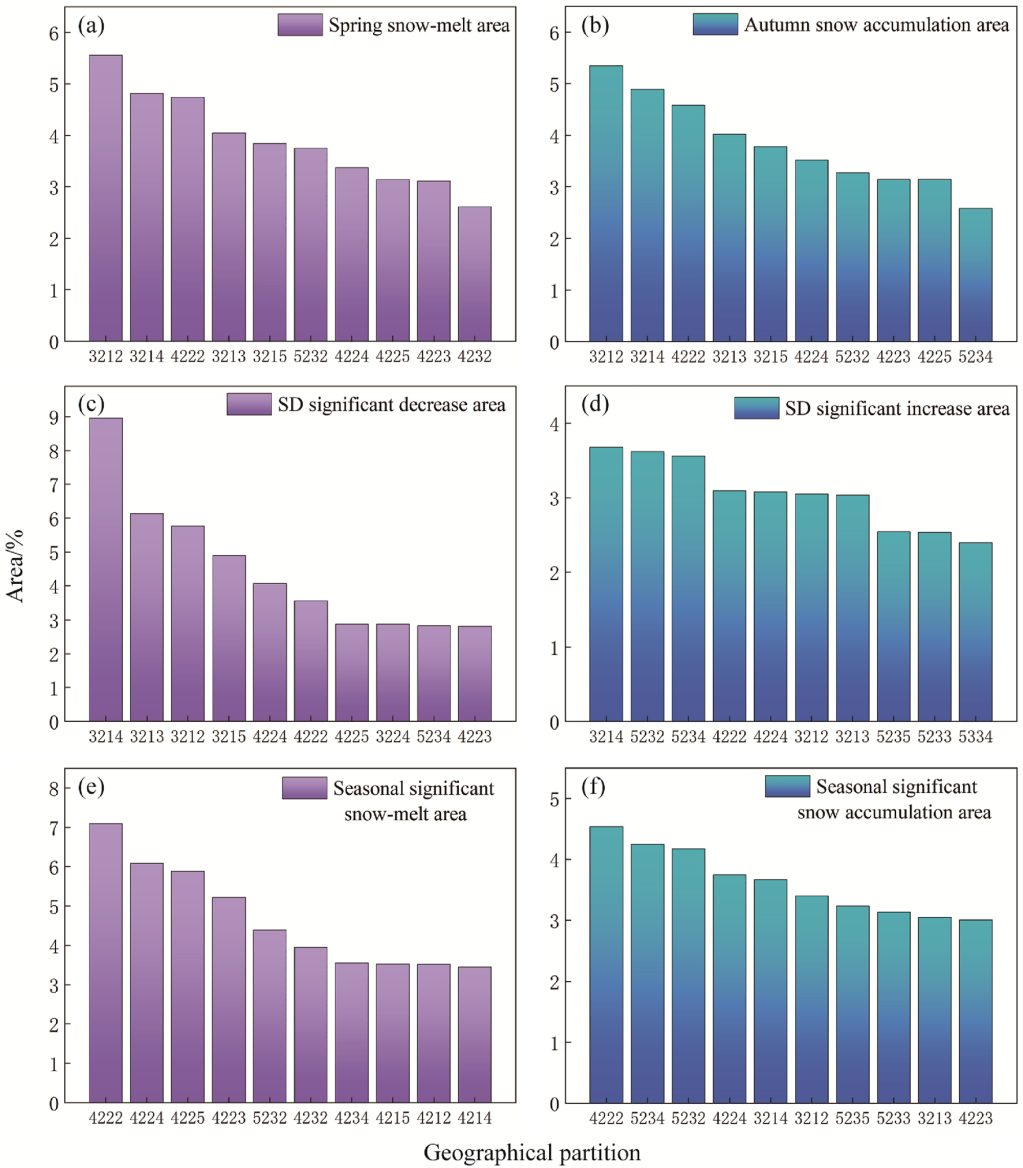
Percentage distribution of geographical partitions area and snow evolution area.

Firstly, according to the characteristics of snow evolution, the Tianshan region is divided into six evolution areas (Fig. 9a–f). Then, the geographical partitions of each evolution area are extracted, and the geographical partition distribution is obtained (Fig. 9g–l). Finally, the area of geographical partitions in each evolution area (Fig. 10a–f) is counted, and the following results are obtained:

1. Seasonal snow evolution area

Spring and autumn are the two seasons with the most obvious seasonal changes in snow cover area. Therefore, seasonal snow evolution is divided into spring snow-melt area (Fig. 9a) and autumn snow accumulation area (Fig. 9b). Figure 9g and h are the distribution of geographical partitions in spring snow-melt area and autumn snow accumulation area, respectively. The area percentage of snow-melt area and study area in spring is 41.12% and the area percentage of snow accumulation area and study area in autumn is 41.12%. The area of the geographical partitions within the statistical area and the percentage of the area of the evolutionary area, ignoring the small area partitions, analyze the distribution of the top ten geographical partitions with larger areas as shown in Fig. 10. The area percentage of the middle altitude hill with shady gentle slope area (3212) in spring snow-melt area and autumn snow accumulation area was the largest (the average GPEP is 5.46%). Because there are many overlapping areas of spring snow-melt area and autumn snow accumulation area, the distribution of geographical partitions in the two areas is similar. It can be seen from the split geographical partition code that the distribution of middle altitude hill (32xx, x is any number corresponding to the position in the geographical partition code) and gentle slope (xx1x) of single geographical factors is more concentrated in the two areas (Fig. 10a and b).

(2) SD significant changes evolution area

There is a significant increase and decrease in SD between years and seasons, Therefore, all the significant increase and all the decrease areas are divided into the SD significant increase and decrease areas (Fig. 9c and d). Figure 9i and j is the distribution of geographical partitions in the two areas. The percentage of SD significant increase and decrease areas to the study area are 15.60% and 7.32%, respectively. The middle altitude hill with sunny and gentle slopes area (3214) has the largest area ratio (GPEP 8.95%) in the two areas, but it shows great difference in single geographical factors. In SD significant decrease area, it is mostly the middle altitude (x2xx) and gentle slope (xx1x) area. In SD significant increase area, it is mostly the geomorphologic type of Middle relief middle altitude mountain (52xx), and the slope is mostly steeper slope.

(3) Seasonal snow significant evolution area

The seasonal snow significant evolution area is the seasonal snow cover evolution area and the snow depth significant evolution intersection area, the evolution of snow cover in the region shows that the change of snow area is active in season, and the snow depth increases or decreases significantly (Fig. 9e and f). Figure 9k shows the distribution of geographical partitions in the seasonal significant snow-melt area and Fig. 9l shows the distribution of geographical partitions in the seasonal significant snow accumulation area. The percentage of area of seasonal significant snow-melt and accumulation area to the study area were 1.90% and 6.49%, respectively. Seasonal snow significant evolution area combines the characteristics of snow cover changes and significant changes in snow depth. The geographical partition with the largest area percentage in the two areas is low relief middle altitude mountain with shady and moderate slope area (4222/GPEP 5.815%), and the single geographical factors in the two areas also show differences. In the seasonal significant snow-melt area, the geomorphologic types are mostly low relief middle altitude mountain, and the slope factors are mostly moderate slope. In seasonal significant snow accumulation area, the geomorphologic types are mostly middle relief middle altitude mountain, and the slope factors are mostly moderate slope and steeper slope.

**Figure 11:**
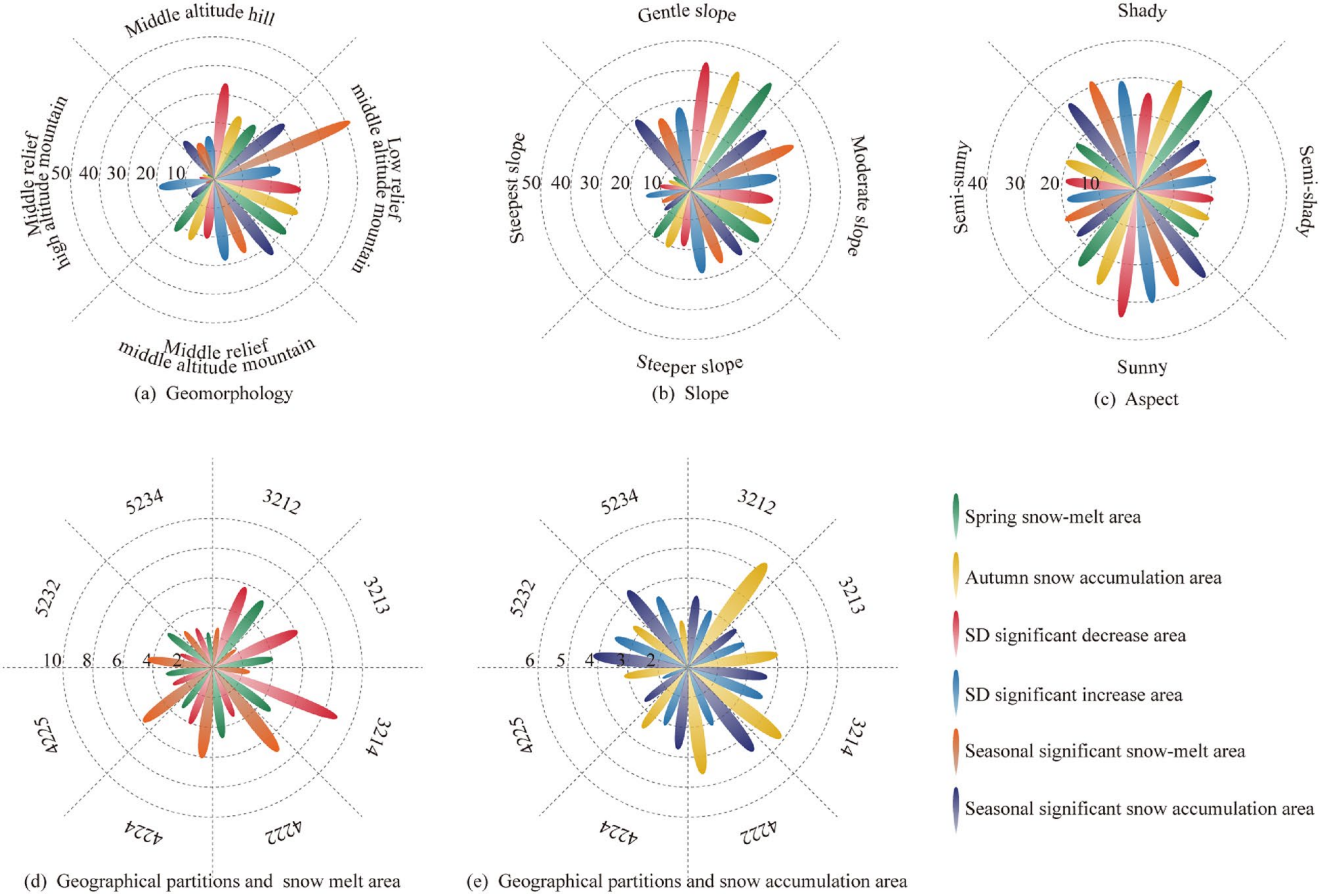
Geographical factors and percentage distribution of evolution area.

**Figure 12:**
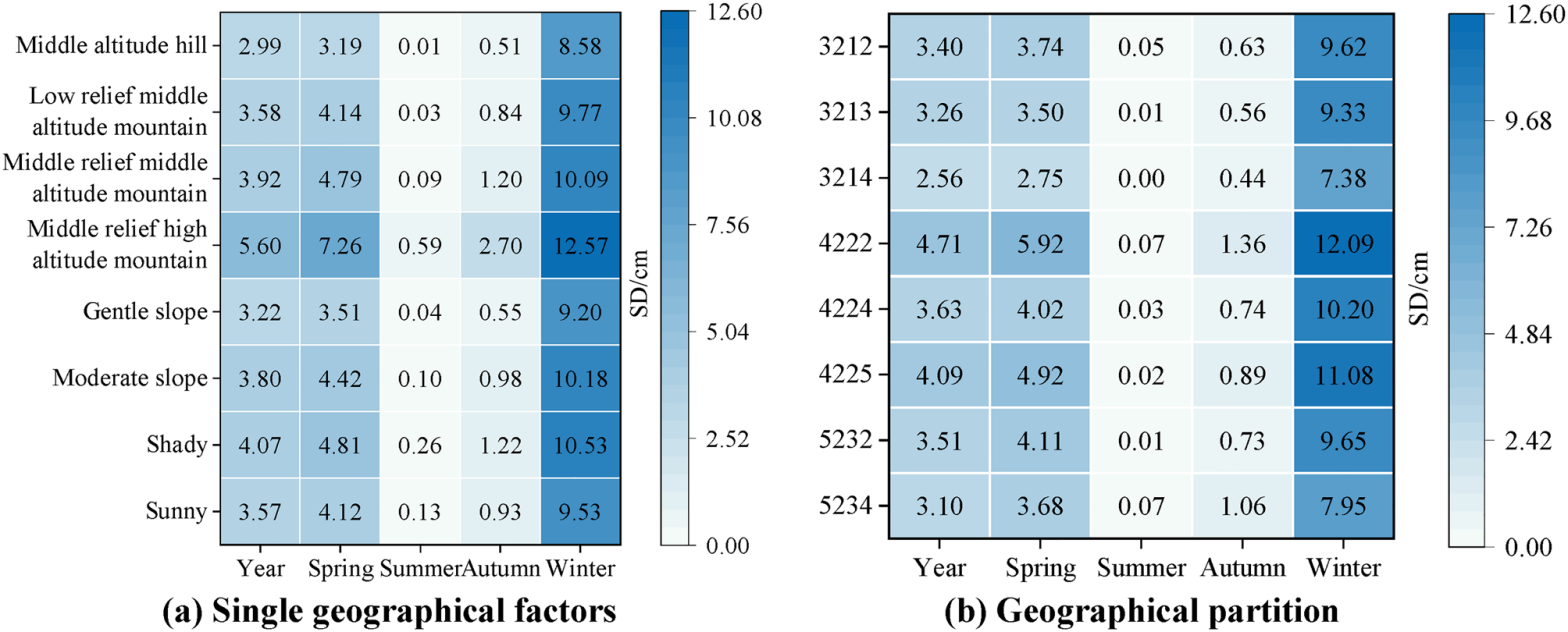
Geographical factors average snow depth year and season.

In the larger geographical partitions in the evolution area, the types of single geographical factors are relatively concentrated, especially the geomorphology and slope factors (Fig. 10). For example, geomorphologic types in the top ten geographical partitions with large areas in Fig. 10 are mainly middle altitude hill (32) and low relief middle altitude mountain (42), slope is mainly gentle and moderate slope. Therefore, the area percentage of single geographical factor in each evolution area is shown in Fig. 11a–c the most active snow evolution on geomorphologic type of low relief middle altitude mountain, with an average GFEP of 33.23%, among the slope types, the snow evolution on gentle slope is the most active, with an average GFEP of 35.49%, among the aspect types, the snow evolution on sunny slope is the most active, with an average GFEP of 29.12%. The variation of snow depth on a single geographical factor with active snow evolution in the study area is shown in Fig. 12a, with the increase of altitude and slope, the snow depth also gradually increases. The geomorphologic type with the largest change in SD is the middle relief high altitude mountain, with a change of 11.98 cm. The slope type with the largest change in SD is the moderate slope, with a change of 10.07 cm. the sunny slope is more likely to receive more solar radiation energy, and the snow is easier to melt. The SD on the sunny slope is smaller than that on the shady slope, and the SD on the shady slope is the largest, with a change of 10.27 cm, the sunny slope is more likely to receive more solar radiation energy, and the snow is easier to melt. Therefore, the SD on the sunny slope is smaller than that on the shady slope, and the SD on the shady slope changes the most, with a variation of 10.27 cm. The snow-melt area (Fig. 11d) is a combination of the spring snow-melt area, the SD significant decrease area and the seasonal significant snow-melt area, the snow accumulation area (Fig. 11e) is a combination of the autumn snow accumulation area, the SD significant increase area and the seasonal significant snow accumulation area. the area of the middle altitude hill with sunny and gentle slopes area (3214) accounted for the largest proportion of the average area of all evolution areas, with an average GPEP of 4.75%. SD changes in geographical partitions with active snow evolution in the study area are shown in Fig. 12b, the low relief middle altitude mountain with shady and moderate slope area (4222) SD changes the most, with a change of 12.02 cm.

**Figure 13:**
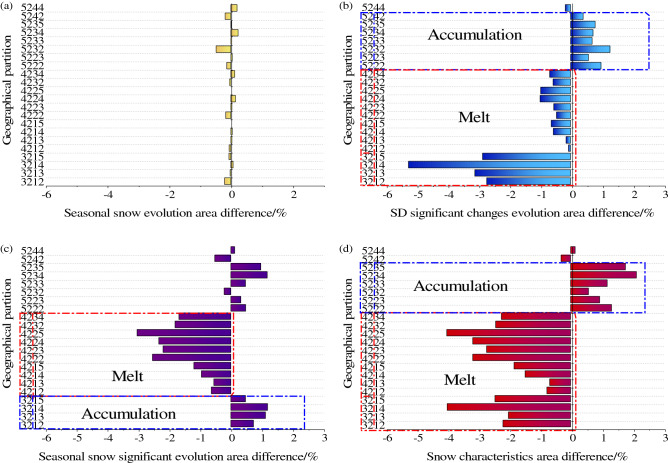
The evolutionary characteristics of snow in 22 geographical partitions of class I.

In order to analyze the characteristics of snow accumulation and snow melting in geological divisions, 6 evolution divisions were divided into 2 parts according to the characteristics of snow evolution. One part is the spring snow-melt area, SD significant decrease area and seasonal significant snow-melt area with snow melting characteristic. The other part is the autumn snow accumulation area, SD significant increase area and seasonal significant snow accumulation area with snow accumulation characteristic. The percentage of the geological partition area of the two parts is used as the classification variable to divide the K-mean into the snow accumulation characteristic category and the snow melting characteristic category in SPSS. The probability of the distance difference between the two categories is less than 0.001, and there are class I and class II geographical partitions in each category. The geographical partition in class I is characterized by a larger proportion of the area in the snow characteristic area than that in class II, that is, the I geographical partitions are more obvious in snow accumulation and snow melting characteristics (Table 7). Figure 13 is the area difference of various snow evolution areas in 22 geographical partitions in the classification of snow melting characteristics and snow characteristics in class I. The positive value indicates the accumulation characteristics of snow, and the negative value indicates the ablation characteristics of snow.

**Table 7:** Cluster centers.

Characteristic: Snowmelt	I (n = 24)	II (n = 262)	Sig
Spring snow-melt area	2.79	0.13	0.000
SD significant decrease area	3.05	0.10	0.000
Seasonal significant snow-melt area	3.29	0.08	0.000
Characteristic: Snow accumulation	I (n = 25)	II (n = 261)	Sig
Autumn snow accumulation area	2.67	0.13	0.000
SD significant increase area	2.40	0.15	0.000
Seasonal significant snow accumulation area	2.86	0.11	0.000

Figure 13a is the difference between the autumn snow accumulation area and the spring snow-melt area in the class I geographical partitions. Because the snow in the spring snow-melt area and the autumn snow accumulation area is almost completely melted in spring and snow will fall in autumn. Class I geographical partitions did not have relatively prominent snow melting and snow accumulation characteristics in this area. Figure 13b is the difference between SD significant increase area and SD significant decrease area. The snow depth of geographical partitions (321x) with gentle topographic relief and slope shows obvious ablation characteristics, while the snow depth of geographical partitions (522x/523x) with large topographic relief and slope shows accumulation characteristics. Figure 13c is the difference between the seasonal significant snow accumulation area and the seasonal significant snow-melt area. In geographical partitions with the geomorphology type middle altitude hill (32xx) shows accumulation characteristics, and in geographical partitions with the geomorphology type low relief middle altitude mountain (42xx) shows snow melting characteristics. Figure 13d is the difference between the evolution area of snow accumulation characteristics and the evolution area of snow melting characteristics. The geographical partitions characteristics of snow melting characteristics are the gentle slope area with the geomorphologic type of middle hill altitude and the gentle slope, moderate slope and steeper slope area with the geomorphologic type of low relief middle altitude mountain. The geographical partitions characteristics of snow accumulation are the moderate slope and steeper slope areas with the geomorphologic type of middle relief middle altitude mountain. On the whole, the elevation characteristics of the 22 class I geographical partitions are all middle altitude (1000–3500 m). The most obvious snow melting is in the gentle slope area of the low topographic relief geomorphology types, and the obvious snow accumulation is in the steep slope area of the middle relief geomorphology types.

## Discussion

Several studies on the evolution of snow cover in alpine regions have shown that topographic factors such as elevation, slope, and aspect have important effects on the accumulation and redistribution of snow cover and increase the temporal and spatial differences of snow cover^26,39,40^. The evolution characteristics of snow cover at different altitudes are obviously different. The low temperature in high altitude area provides favorable conditions for snow accumulation, while the range of snow cover in low altitude area varies greatly^41–44^. The SCP of the whole Tianshan region changes with the seasons, the highest in winter, the snow begins to melt in spring, the snow begins to accumulate in autumn, and there is almost no snow in summer. In the middle and low altitudes, the SCP fluctuates greatly, and the fluctuation of SCP in the high altitude area decreases (Fig. 5). The evolution of snow cover is the most active in Low relief middle altitude mountain, and the evolution of snow cover is the most stable in Middle relief high altitude mountain, The same as the altitude distribution of SCP in the high altitude area of Tianshan region reported by Zhang^45^. There is also a strong correlation between the variation of snow cover and the slope gradient. The snow depth increases with the increase of slope snow depth increases as the slope increases^46–48^. The same trend is observed in the Tianshan region, where the snow cover is more variable on gentle slope areas (Fig. 11b), and the annual and seasonal average snow depth are significantly greater on moderate slope than on gentle slope (Fig. 12a). The aspect affects the intensity of solar radiation received by snow cover. In the Northern Hemisphere, the sunny aspect (mainly the southern aspect) receives sunlight, and the falling snow will melt rapidly, while the shady aspect (mainly the northern aspect) receives less solar radiation, which is often more humid cold than the sunny aspect^49,50^. The Tianshan region are dominated by shady and sunny aspect, and the evolution of snow cover on sunny aspect is more active (Fig. 11c). The annual and seasonal average snow depth on shady aspect is larger than that on sunny aspect (Fig. 12a).

Most of the previous studies^51–54^ analyzed the variation of snow parameters such as snow cover, snow depth and snow water equivalent on the factors of elevation, slope and aspect. Comprehensive evaluation of the influence of geographical factors on snow cover is of great significance for determining snow cover activities^47^. Therefore, this paper extracts the regional distribution of snow cover evolution on all geographical factors, The middle altitude hill with shady and gentle slopes area (3212) has the largest snow melt extent in spring and the largest snow accumulation extent in autumn, and the snow cover variation in this area is the most obvious. The areas with significant increase and decrease of snow depth are the most in the middle altitude hill with sunny and gentle slopes area (3214). In the middle altitude hill area at gentle slope, the shady aspect has the greatest influence on the change of snow cover area, and the sunny aspect has the greatest influence on the increase and decrease of snow depth.

## Conclusion

Based on the superposition of three kinds of geographical factors of Geomorphology, slope and slope aspect in Tianshan region, the geographical zoning of Tianshan region is obtained in this paper. According to the characteristics of the SCP/SD, the study area is divided into spring snow-melt area, autumn snow accumulation area, the SD significant decrease area and the SD significant increase area. Based on the above four snow evolution areas, the seasonal significant snow-melt area and the seasonal significant snow accumulation area are further divided. The geographical partitions in each region are extracted to obtain the geographical characteristics of six evolutionary regions. After the above steps, the following conclusions are obtained:

1. From 2003 to 2017, SCP fluctuated and decreased at the rate of − 0.004 and SD fluctuated and increased at the rate of 0.03. From 2003 to 2017, SCP decreased at the rates of − 0.003, − 0.01 and − 0.025 in spring, summer and winter respectively, SCP increased at the rate of 0.02 in autumn, and SD increased at the rates of 0.0674, 0.008, 0.018 and 0.021 in four seasons. Among the four seasons, the SCP (7.40%) and SD (9.83 cm) are the largest in winter, the SCP (0.7%) and SD (0.15 cm) are the smallest in summer, and the SD in the SD significant changes evolution area is increase at the rate of 0.07 cm·a^−1^, 0.16 cm·a^−1^, 0.05 cm·a^−1^, 0.05 cm·a^−1^, and decrease at the rate of − 0.09 cm·a^−1^ in winter.
2. The largest proportion of single geographical factor area in Tianshan region was middle altitude hill (21.05%), gentle slope (41.83%) and sunny slope (29.45%), the largest proportion of geographical partition area was the middle altitude hill with sunny and gentle slopes area (3214, 4.65%).
3. The snow in the middle altitude hill with shady gentle slope area (3212) is the most obvious in the seasonal evolution, and the percentage of this region in the seasonal snow evolution area is 5.46%, the snow depth in the middle altitude hill with sunny and gentle slopes area (3214) increased and decreased significantly in the past 15 years, and the percentage of this region in the SD significant changes evolution area was 6.32%. The snow in the low relief middle altitude mountain with shady and moderate slope area (4222) not only shows obvious seasonal evolution, but also increases and decreases significantly in snow depth. and the percentage of this region in the seasonal snow significant evolution area is 5.82%.
4. In the single geographical factor type, the area of low relief middle altitude mountain (33.23%), moderate slope (35.49%), sunny aspect (29.12%) accounted for the largest proportion of the average area of all evolution areas, and the area of the middle altitude hill with sunny and gentle slopes area (3214, 4.75%) accounted for the largest proportion of the average area of all evolution areas. The variation of snow depth is the largest in the middle relief high altitude mountain (11.98 cm), the moderate slope (10.07 cm) and the sunny aspect (10.27 cm), and the variation of snow depth is the low relief middle altitude mountain with shady and moderate slope area (4222, 12.02 cm).
5. The snow accumulation and melting are obvious in the range of 1000–3500 m above altitude, different geomorphology types lead to obvious differences in snow characteristics. The snow melting is most obvious in the gentle slope area of the low topographic relief geomorphology types, and the snow accumulation is most obvious in the steep slope area of the middle relief geomorphology types.

## Data Availability

The ASTER Global Digital Elevation Model V003 (ASTER GDEM V3) is available at Earthdata Search (nasa.gov). The Improved MODIS TERRA/AQUA composite Snow and glacier (RGI6.0) data for High Mountain Asia (2002–2018) data is available at https://doi.pangaea.de/10.1594/PANGAEA.901821. The Long-term series of daily snow depth dataset over the Northern Hemisphere based on machine learning (1980–2019) data is available at https://dx.doi.org/10.11888/Snow.tpdc.271701.
